# Burnout in health care workers during the fourth wave of COVID-19: A cross sectional study from Pakistan

**DOI:** 10.1016/j.amsu.2022.104326

**Published:** 2022-08-07

**Authors:** Shoaib Ahmad, Sadia Yaqoob, Sifwa Safdar, Huzaifa Ahmad Cheema, Zarmina Islam, Nida Iqbal, Zoaib Habib Tharwani, Sarya Swed, Mohammad Soban Ijaz, Majeeb Ur Rehman, Abia Shahid, Ufaq Tahir, Shkaib Ahmad, Wajeeha Bilal, Mohammad Yasir Essar, Saleem Iqbal, Zafar Ali Choudry

**Affiliations:** a Punjab Medical College, Faisalabad, Pakistan; b DHQ Teaching Hospital, Faisalabad, Pakistan; c Jinnah Medical and Dental College, Pakistan; d Allama Iqbal Medical College, Lahore, Pakistan; e Department of Medicine, King Edward Medical University, Lahore, Pakistan; f Faculty of Medicine, Dow Medical College, Dow University of Health Sciences, Pakistan; g Mohtarma Benazir Bhutto Shaheed Medical College, Mirpur AJK, Pakistan; h Faculty of Medicine, Aleppo University, Aleppo, Syria; i J.S.S. Medical College, Mysore, India; j Dera Ghazi Khan Medical College, DG Khan, Pakistan; k Kabul University of Medical Sciences, Kabul, Afghanistan; l Department of Surgery, Allied Hospital Faisalabad, Pakistan; m Faisalabad Medical University, Faisalabad, Pakistan

## Abstract

**Objective:**

To assess the burnout among the healthcare workers during the fourth wave of COVID-19.

**Methods:**

In this cross-sectional study, burn out was measured in health care professionals using the MBI scale inventory during the fourth wave of COVID-19. Age, gender, marital status, having children, hospital, job type, experience, and workload, as well as the severity of burnout in each subscale, were all measured. We used the chi-square test to detect the difference between the level of burnout and other demographic variables, and a multiple logistic regression test was used to define the predicted correlation between the high level of burnout and the risk factors. Odds ratios and corresponding 95% confidence intervals (CI) were reported. A p-value of less than 0.05 indicated a statistically significant outcome.

**Results:**

Out of 776 healthcare workers who participated in our study, 468 (63.2%), 161 (21.7%) and 112 (15.1%) participants experienced low, moderate and high levels of emotional exhaustion, respectively. For the depersonalization subscale, 358 (48.3%), 188 (25.4%) and 195 (26.3%) people suffered from low, moderate, and high levels of depersonalization, respectively while 649 (87.6%), 40 (5.4%) and 52 (7.0%) respondents had low, moderate and high levels in the personal accomplishment subscale, respectively.

**Conclusion:**

During the fourth wave of COVID-19, the healthcare workers reported increased level of burnout overall possibly due to the long term physical and mental impacts that the pandemic has had over the time. Moreover, healthcare workers in Pakistan were more prone to burnout as compared to other countries.

## Highlights

• Higher levels of burnout were observed in the fourth wave of COVID-19, possibly due to the long term physical and mental impacts that the pandemic has had over time.
• Healthcare workers in Pakistan were more prone to burnout as compared to other countries.
• This study yielded key findings that have important implications for preventing and reducing burnout in hospital settings.

## 1 Introduction

Work-related stress syndrome, better known as burnout, is defined as over exhaustion characterized by an emotional and psychological component that can severely impact workplace efficacy [1]. Although it occurs in all job settings with a prevalence of up to 20%, physicians are particularly susceptible given their working conditions [1]. For example, the 2020 Medscape National Physician Burnout and Suicide Report reported a burnout prevalence of 43%, whereas another study presents this at 37.9% compared to the control (27.8%) [1]. In addition, a recent systematic review reports a range of 10–59% [2]. With the onset of the COVID-19 pandemic, frontlines have been afflicted with various stressors such as violence, a high emotional burden, fatigue from long working hours, fear of infection, isolation from family members, and chronic stress, which contribute to the increasing prevalence of burnout [1]. With other impacts of COVID-19 like social isolation, disruption of daily routine, the experience of losing a loved one to COVID-19, and financial difficulties, Healthcare workers (HCWs) are even more vulnerable to burnout. Furthermore, predisposing conditions of middle or low-income countries such as socioeconomic disparities, poor social safety nets, weak healthcare systems, reduced healthcare capacity, and political instability can exacerbate stress levels. For example, Pakistan, a developing nation with primary healthcare challenges, reports more burnout among its HCWs [3].

Anxiety levels have increased with the entrance of the fourth wave of the COVID-19 pandemic in Pakistan, and with the predominance of the Delta variant, anxiety levels have increased [4]. In the second wave, a regression analysis evaluating Generalized Anxiety Disorder and depressive symptoms in 500 participants found that 25.4% had GAD and 18.8% had depressive symptoms indicating severe psychological impacts of the pandemic [5]. As of August 2021, 1,160,119 confirmed COVID-19 cases and 25,788 deaths had been reported, with a positivity rate of 6.78% [6]. However, only 13% of the population is fully vaccinated in Pakistan [6]. Unvaccinated patients experience more severe outcomes following infection with COVID-19, as reported by a study of 884 participants during the fourth wave from a tertiary care hospital in Karachi, Pakistan [4]. In this study, the effect of COVID-19 was critical in 10% of unvaccinated patients [4]. This is compounded by increasing Delta variants and slow vaccination rate, increasing the burden on HCWs, thus exacerbating stress levels [7].

High levels of burnout among HCWs existed in Pakistan even before the pandemic. A study of 106 nurses at a tertiary care hospital in Lahore finds a prevalence of burnout in 79% of participants [3]. At the same time, another cross-sectional focusing on burnout among 179 physicians working in emergency departments reports 42.4% for emotional exahustion [8]. A 2019 study assessing 118 surgical residents in Karachi finds higher emotional fatigue in females (49.2%) in comparison to males (50.8%), identifies high levels of sleep deprivation, and a higher personal fulfilment score in married (in contrast to living alone) [9].

In the first wave of COVID-19, a study across six hospitals in Pakistan of 400 HCWs assessed on the PHQ-9 and GAD-7 scale found that 21.8% reported moderate-severe anxiety or depression [10]. Similarly, findings from a study of 398 HCWs from Punjab report a prevalence of 21.4% and 21.9% for anxiety and depression, respectively [11]. In the second wave of COVID-19, a study of 87 physicians reported emotional exhaustion in 54% of participants, depersonalization in 77%, and low personal accomplishment in 31% of participants – all of which were associated with a history of COVID-19 infection or interaction with such patients [12]. Since burnout has short- and long-term implications such as poor delivery of quality healthcare, increased errors, lower quality of life, poor job satisfaction, and substantial costs, it is essential to understand this within a lower-middle-income country like Pakistan [1]. However, the prevalence of burnout in HCWs has not been frequently reported in Pakistan during the fourth wave of the pandemic. Hence, our study aims to assess levels of burnout among HCWs in Pakistan amidst the fourth wave of the pandemic and find any significant associations with age, gender, or care for COVID-19 patients.

## 2 Methods

### 2.1 Study design, setting and participants

This study is cross-sectional about burnout among health care professionals in the fourth wave of COVID-19 in Pakistan. The study was conducted during the fourth wave of COVID-19 in Pakistan that started in July 2021 and included only healthcare professionals with COVID-19 patients contact from all specialties. The study depended on a convenience sampling type for data collection. Raosoft sample size calculator was used to calculate minimum sample size with a confidence interval of 95%. The online self-questionnaire in English was shared on social media platforms such as WhatsApp, Facebook, and telegram to collect a suitable number of respondents. We used a structured self-administered questionnaire which was developed based on a published study about the same topic [13] in the literature. Then it was updated to make it more appropriate according to the population. We performed a pilot study on 30 health care professionals to assess their understanding of the questionnaire's questions to clarify them. The first page of the online survey included a question for acceptance in completing the survey. In addition, the work has been reported in the line with the STROCSS criteria [14].

### 2.2 Measures

The questionnaire consisted of two sections and 36 questions:

● Demographic details include age, gender, marital status, number of children, job title, location of employment, and years of experience. This part also inquired if the responder has dealt with COVID-19 patients and, if so, for how many shifts per month and how many hours each shift.
● Validated tool for measuring the severity of burnout during COVID-19 pandemic: It includes three subscales related to dimensions of emotional exhaustion, depersonalization, and personal accomplishment. The tool consists of 22 questions evaluated on a five-point Likert scale. Every subscale score is computed by adding all of the values from all of the components in that subscale, with the hypothesis that the components in the life satisfaction domain are scored in a reverse way. Emotional fatigue scores range from 0 to 36, depersonalization scores from 0 to 20, and personal accomplishment scores from 0 to 32. Conventional cut-off ratios were used to describe low, moderate, and high levels for each measure. We defined "high burnout" as a moderate or high level on either the physical fatigue or depersonalization dimensions, typically called the "core" of burnout.

### 2.3 Ethical consideration

We obtained ethical approval form Faisalabad Medical University for conducting our research on health care professionals. Each participant had agreed to contribute to completing the questionnaire prior to participation. The contribution to this research was voluntary, and all individual details were saved by a confidential method by collecting anonymous responses.

### 2.4 Statistical analysis

The authors carefully inserted data from the hard copy surveys into the original Google Form online questionnaire used to collect online data. Then the data was exported automatically from Google Form into an Excel format. The raw data was then encrypted in the Excel sheet to be used with the statistics program. We used Statistical Package for Social Sciences version 28.0 (SPSS Inc., Chicago, IL, United States) to perform statistical analysis. Categorical baseline variables were presented as frequencies and percentages, while continuous variables were assigned as means and standard deviations (SDs). We used the chi-square test to detect the difference between the level of burnout and other demographic variables, and a multiple logistic regression test was used to define the predicted correlation between the high level of burnout and the risk factors. Odds ratios and corresponding 95% confidence intervals (CI) were reported. A p-value of less than 0.05 indicated a statistically significant outcome.

## 3 Results

A total of 776 healthcare workers participated in our survey out of which 35 (4.7%) were excluded due to incompletely filled questionnaires. The baseline characteristics and mean Maslach burnout inventory (MBI) scores in each subscale of the participants are given in Table 1. Over half of our participants (57.4%) declared that they had not taken care of COVID-19 patients.

**Table 1:** Baseline characteristics of participants (n = 741).

Variable	Value
**Age in years, Mean ± SD**	27.20 ± 8.25
**Gender, n (%)**	
Female	350 (47.2)
Male	388 (52.4)
Prefer not to say	3 (0.4)
**Marital status, n (%)**	
Engaged/in a relationship	46 (6.2)
Married	198 (26.7)
Separated/Divorced	2 (0.3)
Single	492 (66.4)
Widowed	3 (0.4)
**Having children, n (%)**	
Yes	160 (21.6)
No	581 (78.4)
**Province, n (%)**	
Baluchistan	18 (2.5)
KPK	93 (12.8)
Punjab	458 (63.1)
Sindh	157 (21.6)
**Job category, n (%)**	
Specialist	114 (15.4)
Resident	137 (18.5)
Intern (House Officer)	288 (38.9)
Nurse	87 (11.7)
Other	115 (15.5)
**Years in practice, Mean ± SD**	4.56 ± 6.41
**Average daily workload, Mean ± SD**	5.25 ± 4.31
**Cared for COVID-19 patients, n (%)**	
Yes	316 (42.6)
No	425 (57.4)
**Maslach Burnout Inventory Score, Mean ± SD**	
Total score	43.20 ± 18.90
Emotional Exhaustion Subscale Score	15.20 ± 11.70
Depersonalization Subscale Score	7.68 ± 6.88
Personal Accomplishment Subscale Score	20.30 ± 12.3

*n* number, *SD* standard deviation, *COVID-19* corona virus disease 2019.

In our study, 468 (63.2%), 161 (21.7%) and 112 (15.1%) participants experienced low, moderate and high levels of emotional exhaustion, respectively. For the depersonalization subscale, 358 (48.3%), 188 (25.4%) and 195 (26.3%) people suffered from low, moderate and high levels of depersonalization, respectively while 649 (87.6%), 40 (5.4%) and 52 (7.0%) respondents had low, moderate and high levels in the personal accomplishment subscale, respectively.

Table 2 demonstrates the association between the different subscales and the sociodemographic characteristics of the participants. The province of residence was the only variable significantly associated with all the three subscales with participants from Baluchistan experiencing higher levels of burnout.

**Table 2:** Participant's level of burnout in each dimension by sociodemographic characteristics and job category, number (%).

	Emotional Exhaustion				Depersonalization				Personal Accomplishment			
	Low	Moderate	High	*P*	Low	Moderate	High	*P*	Low	Moderate	High	*P*
Age												
≤36 year	398 (61.8)	147 (22.8)	99 (15.4)	0.112	289 (44.9)	169 (26.2)	186 (28.9)	**< 0.001**	563 (87.4)	35 (5.4)	46 (7.1)	0.723
>36 year	70 (72.2)	14 (14.4)	13 (13.4)		69 (71.1)	19 (19.6)	9 (9.3)		86 (88.7)	5 (5.2)	6 (6.2)	
**Gender**												
Female	214 (61.1)	80 (22.9)	56 (16.0)	0.289	180 (51.4)	93 (26.6)	77 (22.0)	0.068	307 (87.7)	18 (5.1)	25 (7.1)	0.488
Male	253 (65.2)	79 (20.4)	56 (14.4)		177 (45.6)	95 (24.5)	116 (29.9)		340 (87.6)	22 (5.7)	26 (6.7)	
**Marital status**												
Married	124 (62.6)	40 (20.2)	34 (17.2)	0.711	116 (58.6)	46 (23.2)	36 (18.2)	**0.005**	178 (89.9)	11 (5.6)	9 (4.5)	0.843
Single	310 (63.0)	113 (23.0 )	69 (14.0)		220 (44.7)	132 (26.8)	140 (28.5)		428 (87.0)	26 (5.3)	38 (7.7)	
Engaged/in a relationship	29 (63.0)	8 (17.4)	9 (19.6)		18 (39.1)	9 (19.6)	19 (41.3)		38 (82.6)	3 (6.5)	5 (10.9)	
Separated/divorced	2 (100.0)	0 (0.0)	0 (0.0)		1 (50.0)	1 (50.0)	0 (0.0)		2 (100.0)	0 (0.0)	0 (0.0)	
Widowed	3 (100.0)	0 (0.0)	0 (0.0)		3 (100.0)	0 (0.0)	0 (0.0)		3 (100.0)	0 (0.0)	0 (0.0)	
**Having children**												
Yes	103 (64.4)	29 (18.1)	28 (17.5)	0.362	98 (61.3)	35 (21.9)	27 (16.9)	**0.001**	144 (90.9)	8 (5.0)	8 (5.0)	0.502
No	365 (62.8)	132 (22.7)	84 (14.5)		260 (44.8)	153 (26.3)	168 (28.9)		505 (86.9)	32 (5.5)	44 (7.6)	
**Province**												
Balochistan	9 (50.0)	1 (5.6)	8 (44.4)	**0.033**	5 (27.8)	5 (27.8)	8 (44.4)	**0.010**	13 (72.2)	3 (16.7)	2 (11.1)	**0.019**
KPK	60 (64.5)	21 (22.6)	12 (12.9		37 (39.8)	22 (23.7)	34 (36.6)		78 (83.9)	10 (10.8)	5 (5.4)	
Punjab	290 (63.3)	106 (23.1)	62 (13.5)		213 (46.5)	122 (26.6)	123 (26.9)		397 (86.7)	23 (5.0)	38 (8.3)	
Sindh	101 (64.3)	30 (19.1)	26 (16.6)		94 (59.9)	36 (22.9)	27 (17.2)		146 (93.0)	4 (2.5)	7 (4.5)	
**Job category**												
Intern (House Officer)	164 (56.9)	79 (27.4)	45 (15.6)	0.122	121 (42.0)	89 (30.9)	78 (27.1)	**< 0.001**	268 (93.1)	9 (3.1)	11 (3.8)	**< 0.001**
Nurse	59 (67.8)	15 (17.2)	13 (14.9)		60 (69.0)	17 (19.5)	10 (11.5)		75 (86.2)	7 (8.0)	5 (5.7)	
Resident	86 (62.8)	29 (21.2)	22 (16.1)		61 (44.5)	40 (29.2)	36 (26.3)		120 (87.6)	9 (6.6)	8 (5.8)	
Specialist	77 (67.5)	22 (19.3)	15 (13.2)		73 (64.0)	23 (20.2)	18 (15.8)		101 (88.6)	5 (4.4)	8 (7.0)	
Other	82 (71.3)	16 (13.9)	17 (14.8)		43 (37.4)	19 (16.5)	53 (46.1)		85 (73.9)	10 (8.7)	20 (17.4)	
**Cared for COVID-19 patients**												
Yes	185 (58.5)	77 (24.4)	54 (17.1)	0.080	158 (50.0)	78 (24.7)	80 (25.3)	0.728	286 (90.5)	17 (5.4)	13 (4.1)	**0.028**
No	283 (66.6)	84 (19.8)	58 (13.6)		200 (47.1)	110 (25.9)	115 (27.1)		363 (85.4)	23 (5.4)	39 (9.2)	

*n* number, *COVID-19* corona virus disease 2019.

Table 3 illustrates the distribution of high burnout in different groups of participants. Overall, the frequency of high burnout in our study was 61.8% (458 out of 741 participants). High levels of burnout were significantly more common in participants less than 36 years old and in those with less than 5 years in practice. A significantly higher proportion of nurses had low levels of burnout as compared to the other professions. Caring for COVID-19 patients and average daily workload were not associated with high levels of burnout.

**Table 3:** Frequency of high burnout in different groups of participants (n = 741).

Variable	High burn out level, n (%)		*P*-value
	Yes	No	
Age			
≤36 year	418 (64.9)	226 (35.1)	<0.001
>36 year	40 (41.2)	57 (58.8)	
**Gender**			
Female	214 (61.1)	136 (38.9)	0.929
Male	242 (62.4)	146 (37.6)	
**Marital status**			
Married	111 (56.1)	87 (43.9)	0.052
Single	315 (64.0)	177 (36.0)	
Engaged/in a relationship	31 (67.4)	15 (32.6)	
Separated/divorced	1 (50.0)	1 (50.0)	
Widowed	0 (0.0)	3 (100.0)	
**Having children**			
Yes	85 (53.1)	75 (46.9)	**0.013**
No	373 (64.2)	208 (35.8)	
**Province**			
Balochistan	14 (77.8)	4 (22.2)	**0.018**
KPK	64 (68.8)	29 (31.2)	
Punjab	291 (63.5)	167 (36.5)	
Sindh	81 (51.6)	76 (48.4)	
**Job category**			
Intern (House Officer)	195 (67.7)	93 (32.3)	**< 0.001**
Nurse	38 (43.7)	49 (56.3)	
Resident	90 (65.7)	47 (34.3)	
Specialist	57 (50.0)	57 (50.0)	
Other	78 (67.8)	37 (32.2)	
**Years in practice**			
≤5 years	374 (65.3)	199 (34.7)	**< 0.001**
>5 years	84 (50.0)	84 (50.0)	
**Cared for COVID-19 patients**			
Yes	192 (60.8)	124 (39.2)	0.647
No	266 (62.6)	159 (37.4)	
**Average daily workload**			
≤ 6 h	112 (60.9)	72 (39.1)	
> 6 h	63 (60.6)	41 (39.4)	0.896

*n* number, *COVID-19* corona virus disease 2019.

^a^only available for those who cared for COVID-19 patients.

The results of multiple logistic regression to identify independent predictors of high burnout levels are presented in Table 4. Having children was associated with an increased risk of high burnout level (OR 3.98, 95% CI: 1.37–11.54). The rest of the variables were not associated with this outcome.

**Table 4:** Predictors of high level of burnout by multiple logistic regression.

Variable	OR	95% CI for OR		*P*-value
		Lower limit	Upper limit	
**Age**	1.00	0.93	1.08	0.978
**Gender**				
Female	1			
Male	1.24	0.71	2.15	0.449
**Marital status**				
Single	1			
Married	0.68	0.29	1.62	0.384
Engaged/in a relationship	1.03	0.34	3.14	0.959
**Having children**				
No	1			**0.011**
Yes	3.98	1.37	11.54	
**Job category**				
Intern (House Officer)	1			
Nurse	0.47	0.20	1.10	0.081
Resident	1.01	0.48	2.13	0.980
Specialist	0.58	0.22	1.57	0.284
Other	2.05	0.50	8.39	0.320
**Years in practice**	0.94	0.86	1.03	0.207
**Cared for COVID-19 patients**				
No	1			
Yes	1.80	0.48	6.72	0.384
**Average daily workload**	1.03	0.97	1.10	0.280

*OR* odds ratio, *CI* confidence interval, *COVID-19* corona virus disease 2019.

## 4 Discussion

Health care professionals continue to buckle under the load as the fourth wave of COVID-19 hits Pakistan. The present study showed a remarkable increment in burnout levels of 61.8%. Our findings are unique in revealing high levels of burnout among healthcare professionals who provided clinical care to non-COVID-19 patients, with substantial variation across different provinces of Pakistan. However, a cross-sectional survey in Italy reported that those taking care of COVID-19 patients were more likely to stay burnout [13]. By the time the fourth wave hit Pakistan, HCWs seemed more prepared to combat the deadly virus, having experience working during the previous three waves, and therefore showed lower levels of burnout than those working in non-COVID units. Hospitals should consider recruiting and hiring an additional healthcare workforce to cater to critically ill, not necessarily COVID patients. This may help to reduce the likelihood of psychological anguish and emotional weariness.

The incidence of burnout level among HCWs is higher, 61.8%, compared to other countries in Asia such as India (52.8%) [15], China (34.2%) [16], and Japan (31.4%) [17] but lower than that in Saudi Arabia (75%) [18]. The differences in demographics, job category, working environment, and average daily workload of participants may account for the diversity of burnout prevalence. Nevertheless, more than half of the frontline warriors were exhausted, demanding urgent needs of comprehensive management strategies for reducing stress.

Our study shows that participants with less than five years of practice had a higher level of burnout (65.3%), which is in coherence with another study which shows that 58.1% of the population who had less than five years of practice had a high burnout level [23]. This can be explained because, over time, the staff becomes more familiar with the work environment and learns how to cope with the stress, therefore, having a low burnout level compared to those with low experience.

Women tend to succumb to psychological distress more than males, as observed by Gramaglia et al. in their study [19]. In contrast, male healthcare professionals were the most at risk of exhaustion in our study. Additionally, burnout is rampant among young healthcare workers. While previous studies [20,21] have reported high levels of burnout among nurses and residents, our study demonstrated increased burnout in house officers. Young HCWs, mainly house officers, may take some time to acclimatize to the new environment and work for long hours. This may add up to stress levels during the initial practicing period.

Our analysis found that the majority of the population suffered from low levels of emotional exhaustion (63.2%), depersonalization (48.3%), and personal accomplishment (87.6%). House officers who did not care for COVID-19 patients and residents of Punjab achieved low personal achievement scores. In contrast, a cohort study conducted in Italy showed higher emotional exhaustion and depersonalization and lowered personal accomplishment [22]. The mean scores across emotional exhaustion, depersonalization, and lack of personal accomplishment subscales were much lower (15.2, 7.68, 20.3) than in a study conducted in Tehran during the first wave of COVID-19 when cases were on the rise (26.6, 10.2, and 27.3) [23]. With immense support from UNICEF, there was rapid induction of trained health workforce during the fourth wave of COVID-19 that aided in curtailing psychological stress in various healthcare departments [24].

This study yielded key findings that have important implications for preventing and reducing burnout in hospital settings. In light of the foregoing, the study suggests that healthcare employees' mental health be assessed regularly, with healthcare organizations providing need-based interventions. However, there were certain limitations associated with this study. First, this study used an online cross-sectional survey form, limiting the accessibility of the form to those who do not use social media or the internet. Secondly, even though this study was conducted throughout Pakistan, it was difficult collecting sufficient data from all the provinces; therefore, providing a precise comparison between different provinces was challenging due to a limited number of responses from Sindh, Balochistan, and KPK.

Moreover, participant bias could be involved in portraying a better image of oneself while answering a response sheet which can hamper the results. Sampling bias may also be present because HCWs might have been busy filling out the forms. To counter these limitations, more studies should be conducted in different provinces of Pakistan to provide a better comparison, and Social Desirability Scale, along with other tools, should be used to improve the validity of questionnaire-based research.

## 5 Conclusion

Higher levels of burnout were observed in the fourth wave of COVID-19, possibly due to the long term physical and mental impacts that the pandemic has had over time. Healthcare workers in Pakistan were more prone to burnout as compared to other countries.

## Provenance and peer review

Not commissioned, externally peer-reviewed.

## Ethical approval

We obtained an ethical approval form Faisalabad Medical University for conducting our research on health care professionals.

## Sources of funding

None.

## Author contribution

SA, ME, and SS were responsible for the idea and study design. SA, HAC, and SS performed the data analysis. ZI, SY, SS wrote the first of the manuscript. All other authors collected the data, performed, shared in the writing, formatting, and approval of the final version.

## Registration of research studies

1. Name of the registry:
2. Unique Identifying number or registration ID:
3. Hyperlink to your specific registration (must be publicly accessible and will be checked):

## Guarantor

Shoaib Ahmad.

## Consent

N/A.

## Declaration of competing interest

None declared.
